# Pulse field ablation in patients with paroxysmal and persistent atrial fibrillation using the circular multielectrode array catheter: first outcome data in a real-world prospective study

**DOI:** 10.1007/s10840-025-02234-1

**Published:** 2026-01-14

**Authors:** Verena Tscholl, Toshinori Chiba, Philipp Formum, Peter Wienke, Patrick Nagel, Johannes Lucas, Martin Huemer, Ulf Landmesser, Nikolaos Dagres, Felix Hohendanner, Gerhard Hindricks, Robert Hättasch, Philipp Attanasio

**Affiliations:** 1 Department of Cardiology, Angiology and Intensive Care Medicine, Deutsches Herzzentrum der Charité, Charitéplatz 1, 10117, Berlin, Germany; 2 Department of Cardiology, Angiology and Intensive Care Medicine, Deutsches Herzzentrum der Charité, Hindenburgdamm 30, 12203 , Berlin, Germany

**Keywords:** Pulmonary vein isolation, Pulse field ablation, Atrial fibrillation, Circular multielectrode array catheter

## Abstract

**Background:**

Pulsed field ablation (PFA) is an innovative option for the treatment of atrial fibrillation (AF). As different PFA technologies are now available, the safety and efficacy of each ablation system must be individually demonstrated. For the circular multielectrode array catheter, only the pivotal trial to date has been published showing efficacy and safety comparable to other PFA technologies. This study aimed to evaluate outcomes of this technology in a real-world cohort.

**Methods:**

Consecutive patients undergoing their first pulmonary vein isolation (PVI) using the circular multielectrode array catheter were included. All patients were prospectively enrolled in the Berlin Best-Ablate registry. Follow-up evaluations were performed at 3, 6, and 12 months using 24-hour Holter electrocardiography.

**Results:**

A total of 81 patients (37 with paroxysmal AF and 44 with persistent AF) were included. Acute PVI was achieved in all patients (median age, 69 years; 64.2% male). After a mean follow-up of 6.7 ± 5.1 months, recurrence of any atrial arrhythmia was observed in 23 of 81 patients (28.4%). Four patients (three with atrial tachycardia (AT) and one with AF) underwent a repeat ablation procedure. In both AT cases, PVI was found to be intact, whereas in the AF case, re-isolation of the pulmonary veins was required. One patient experienced a major complication (stroke).

**Conclusion:**

In this prospective real-world study, PFA using the circular multielectrode array catheter (PulseSelect catheter) proved to be effective in patients with both paroxysmal and persistent AF, corroborating the findings of the pivotal trial.

**Graphical Abstract:**

**Figure:**
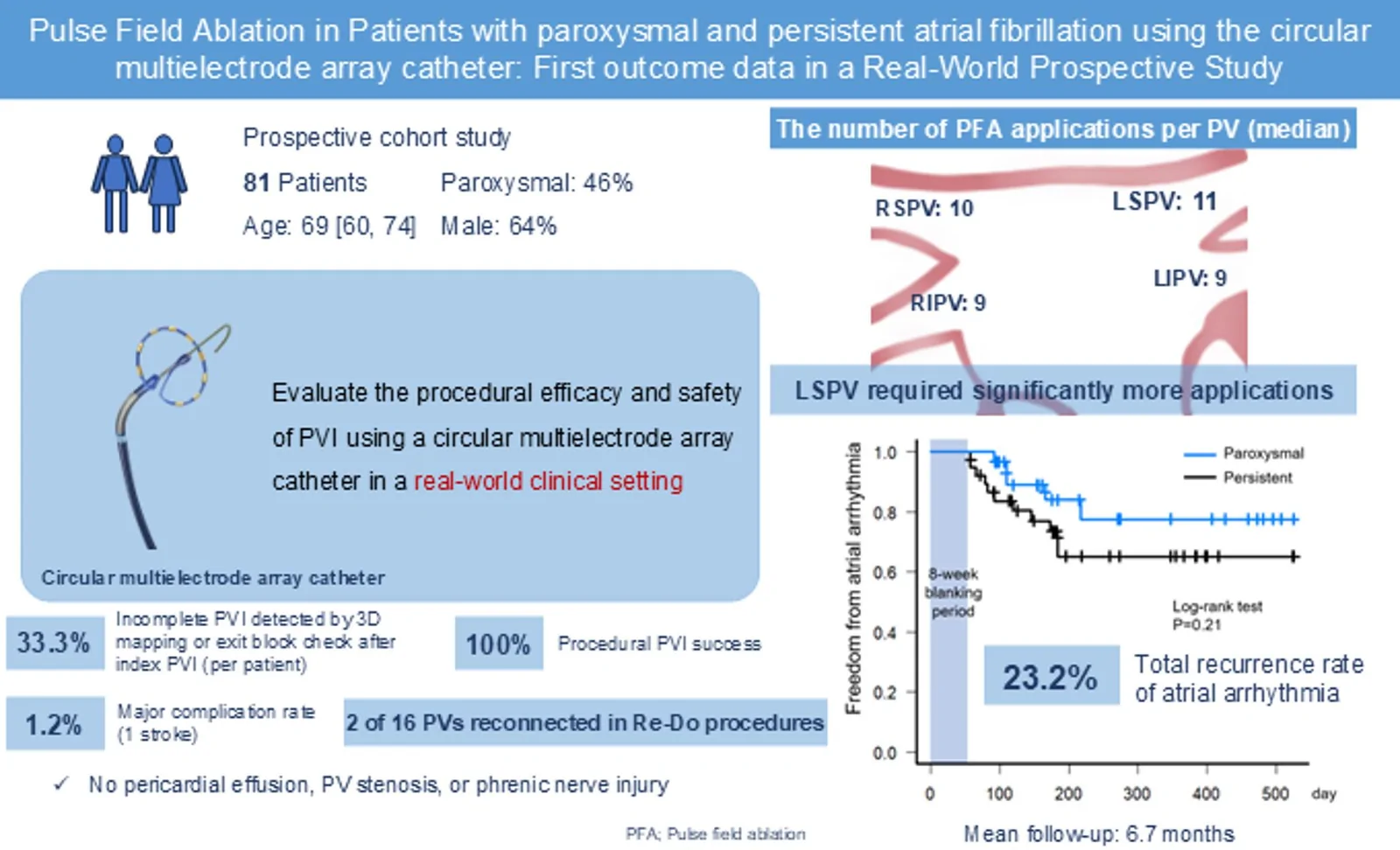

## Background

Pulsed field ablation (PFA) is a novel treatment option for atrial fibrillation (AF). In addition to established techniques such as cryoballoon and radiofrequency ablation, PFA has demonstrated potential as a safe and efficacious strategy for achieving pulmonary vein isolation (PVI) [1]. To date, various PFA technologies using different ablation protocols and catheter designs have been developed and approved for commercial use [2–5] Although safety and efficacy of these platforms have been shown in preliminary trials, real word data is needed to confirm these results in different settings. Safety and efficacy data on using the circular multielectrode array catheter (PulseSelect, Medtronic, Minneapolis, MN) have been published in the PULSED AF pivotal trial [6] and demonstrated a low rate of primary safety adverse events (0.7%) and a similar effectiveness when compared to established ablation technologies. Procedural outcomes of pulmonary vein isolation (PVI) using this catheter have previously been reported; however, no additional data on longer-term outcomes are currently available. The PULSED AF pivotal trial demonstrated favorable safety and efficacy of the PulseSelect system in selected patients with paroxysmal and persistent AF under tightly controlled study conditions. However, pivotal trials typically enroll highly selected populations with protocolized follow-up and may not fully reflect the heterogeneity of real-world AF patients or contemporary workflows in routine practice. Therefore, further independent real-world studies are needed to validate the performance and safety of the PulseSelect catheter across diverse patient profiles and procedural settings and to complement the findings of the pivotal trial. The present study was designed to evaluate the procedural efficacy and safety of PVI performed with the circular multielectrode array catheter, representing the first report of its use in a real-world clinical setting.

## Methods

### Study population

From February 2024 to July 2025, consecutive patients with paroxysmal or persistent AF who underwent first PVI using the circular multielectrode array catheter (PulseSelect Medtronic) were included in this prospective analysis. All patients included in this study were prospectively enrolled in the Berlin Best-Ablate registry and gave written informed consent prior to the inclusion. Patients were eligible for inclusion if they were adults aged 18 years or older and had a diagnosis of atrial fibrillation, either paroxysmal or persistent. The use of anti-arrhythmic drugs prior to ablation was allowed at the discretion of the treating physician. Individuals with a history of atrial tachycardia or other atrial tachyarrhythmias were excluded. Structural heart disease was permitted and was not considered an exclusion criterion. Long-standing persistent atrial fibrillation lasting more than one year was defined as an exclusion criterion. This study was approved by the local ethics committee (EA4/063/15) and conforms to the guiding principles of the Declaration of Helsinki of 2024.

### Ablation procedures

Transesophageal echocardiography was performed in patients not on continuous anticoagulation prior to the ablation procedure to rule out left atrial thrombi. Procedures were performed without DOAC interruption.

All patients received deep sedation with propofol and fentanyl. Venous access was obtained via the femoral vein using ultrasound guidance. A diagnostic catheter was placed in the coronary sinus (Inquiry, 10 Pole, Abbott, Abbott Park, IL). Left atrial access was obtained with a single transseptal puncture using a modified Brockenbrough technique under fluoroscopic guidance with the 10 F steerable sheath (FlexCath Contour, Medtronic, Minneapolis, MN). Unfractionated heparin was administered according to body weight, with a target activated clotting time (ACT) of > 300 s. ACT levels were analyzed every twenty minutes thereafter. If patients were in AF, electrical cardioversion was performed prior to the first ablation pulse. The circular multielectrode array catheter (PulseSelect, Medtronic, Minneapolis, MN) was introduced using a guidewire (PV Tracker, Medtronic, Minneapolis, MN). Before energy delivery, 0.5–1.5 mg Atropine was administered to avoid vagally induced bradycardias. A minimum set of 4 pulses was delivered in the pulmonary vein (PV) ostium and additional 4 pulses at an antral position. After each pulse delivery, the catheter was rotated to achieve full circumferential isolation of the PV. The catheter positioning was guided via fluoroscopy. After these initial applications, additional pulses were delivered when residual pulmonary vein potentials were observed. Following the disappearance of PV potentials, pacing from within the PV was conducted to confirm exit block. PVI was confirmed by demonstrating both entrance and exit block in the PV ostium with the circular catheter. Additional applications were delivered when exit block was incomplete. When residual carina potentials were identified, additional pulses were delivered with careful attention to catheter contact at the carina. However, routine carina-targeted ablation was not performed. In 50 patients, 3D mapping (CARTO 3; Biosense Webster, Irvine, CA) was performed both before and after PVI during sinus rhythm using either a Pentaray (Biosense Webster, Irvine, CA) or a Lasso catheter (Biosense Webster, Irvine, CA). If PVI could not be confirmed on 3D mapping or by exit block assessment after the index PVI, additional PFA pulses were delivered. In March 2025, the procedural protocol was revised to discontinue the use of 3D mapping in order to shorten procedure time and improve cost efficiency. As a result, cases performed after March 2025 did not include 3D mapping, and its use was determined by this protocol change rather than by patient-specific factors. No extra PVI ablation was performed.

### Postprocedural management

The puncture site was closed using a figure-of-eight suture technique, ensuring hemostasis. All patients were observed for 24 h after the procedure with continuous electrocardiographic monitoring. Pericardial effusion was ruled out by transthoracic echocardiography immediately after the procedure and before discharge. Anti-arrhythmic drugs were used before ablation at the discretion of the treating physician. After the procedure, these medications were discontinued either immediately or after the 8-week blanking period, depending on the operator’s judgment. Therefore, all patients discontinued anti-arrhythmic therapy by the end of the blanking period.

### Definitions of complications

Major complications were noted and defined as described in the HRS/EHRA/ECAS Expert Consensus Statement 2024 [7].

### Follow up

Follow up was scheduled at 3, 6 and 12 months, using 24-hour holter electrocardiography monitoring. Symptom-triggered ECG recordings were permitted for patients who experienced palpitations or other symptoms suggestive of arrhythmia recurrence. Atrial Arrhythmia (AA) recurrence was defined as episodes of atrial tachyarrhythmias (ATa) lasting ≥ 30 s. Arrhythmia recurrence was evaluated in two ways: (1) including recurrences during the 8-week blanking period and (2) excluding blanking-period recurrences. The primary analysis assessed arrhythmia-free survival based on recurrences occurring after the blanking period.

### Statistical analysis

The continuous variables are reported as the median [Q1,3]. Continuous variables that did not follow a normal distribution were compared between two independent groups using the Mann–Whitney U test. The AA recurrence rate between paroxysmal and persistent AF was evaluated with the use of a log-rank test. Survival curves were constructed using the Kaplan–Meier estimator according to standard methodology. Patients without documented recurrence were censored at their final available follow-up. P values of < 0.05 were considered statistically significant. The sample size was calculated to estimate the proportion of patients free from atrial fibrillation (AF) at 12 months with a precision of ± 10% points and a 95% confidence interval (CI). Assuming an expected AF freedom rate of 70%, the required sample size was a minimum of 81 patients. The 95% confidence interval for the proportion of PV reconnection was calculated using the Clopper–Pearson exact method to account for the small sample size. All statistics were analyzed using R software version 4.0.3 (R Foundation for Statistical Computing, Vienna, Austria).

## Results

### Study population

81 patients (median age 69 [60,74] years; 64.2% male) with paroxysmal (45.7%) or persistent AF were included in this study. Mean CHA2DS2-VA score and HAS-BLED score were 2 [1, 3] and 1 [1, 1], respectively. Median left atrial (LA) volume index was 41.5 [34.0, 49.0] ml/m^2^, and median left ventricular ejection fraction was 56.0 [50.0, 59.0] %. The patient characteristics are shown in Table 1.

**Table 1:** Characteristics of the total patients. Continuous variables are presented as median [Q1, Q3]

Patient characteristics	*N* = 81
Age, year	69 [60, 74]
Male, n (%)	52 (64.2)
Body mass index, kg/m^2^	27.2 [23.9, 29.7]
CHA2DS2-VA score	2 [1, 3]
HAS-BLED sore	1 [1, 1]
Paroxysmal atrial fibrillation, n (%)	37 (45.7)
Hypertension, n (%)	54 (66.7)
Diabetes, n (%)	12 (14.8)
Chronic kidney disease, n (%)	8 (9.9)
Stroke/transient ischemic attack, n (%)	3 (3.7)
Left ventricular ejection fraction, %	56.0 [50.0, 59.0]
Left atrial volume index, ml/m^2^	41.5 [34.0, 49.0]

### Procedural characteristics

**Fig. 1:**
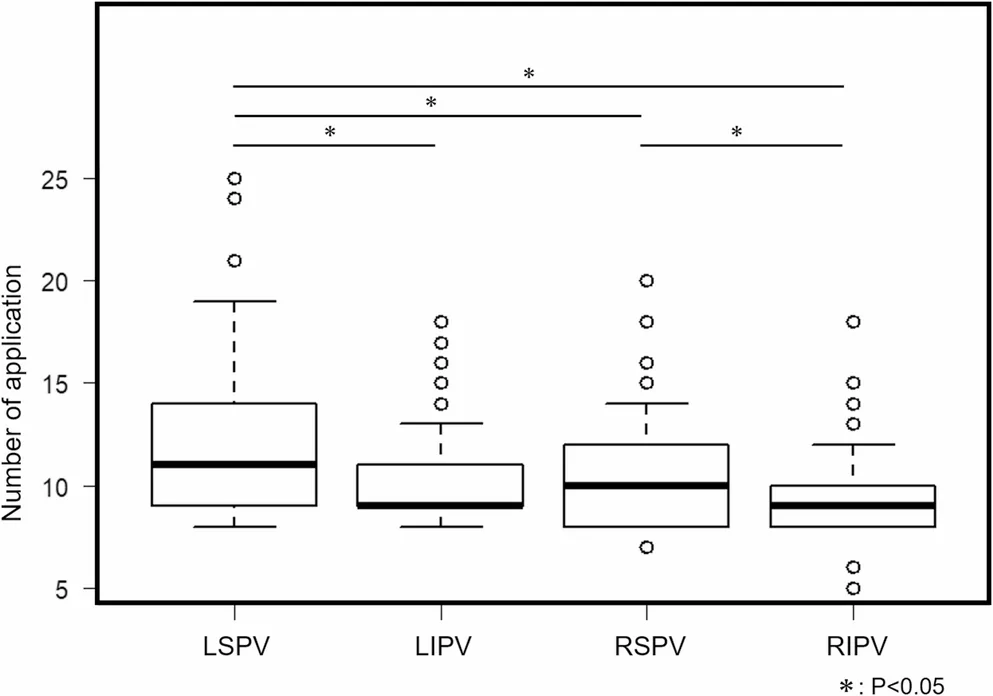
The median number of PFA applications per pulmonary vein for isolation. The LSPV required a significantly higher number of PFA applications compared to the other PVs. LSPV, left superior pulmonary vein; LIPV, left inferior pulmonary vein; RSPV, right superior pulmonary vein; RIPV, right inferior pulmonary vein

Fifty patients (60.2%) underwent post ablation 3D mapping. The median procedural time was 57 [44, 69] minutes (with 3D mapping; 74 min, without 3D mapping; 57 min, *P* < 0.01), and the mean LA dwell time was 55 [45, 63] minutes. The median time from first to last PFA application was 29 [24,34] minutes (Table 2). Acute PVI was achieved in all patients. The mean number of PFA applications per patient for PV isolation was 41 [36, 47]. A significantly higher number of PFA applications was applied at the LSPV compared to the other PVs (the median number of applications, LSPV:11, RSPV:10, RIPV:9, LIPV:9) (Fig. 1).

**Table 2:** Procedural data: continuous variables are presented as median [Q1, Q3]. PV, pulmonary vein

	*N* = 83
Procedure time, minute	68 [59, 82]
Left atrial dwell time, minute	55 [45, 63]
Ablation time, minute	29 [24, 34]
Fluoroscopy time, minute	18.1 [14.4, 22.1]
Mapping time, minute	7 [5.8, 10.0]

### Clinical success rate

**Fig. 2:**
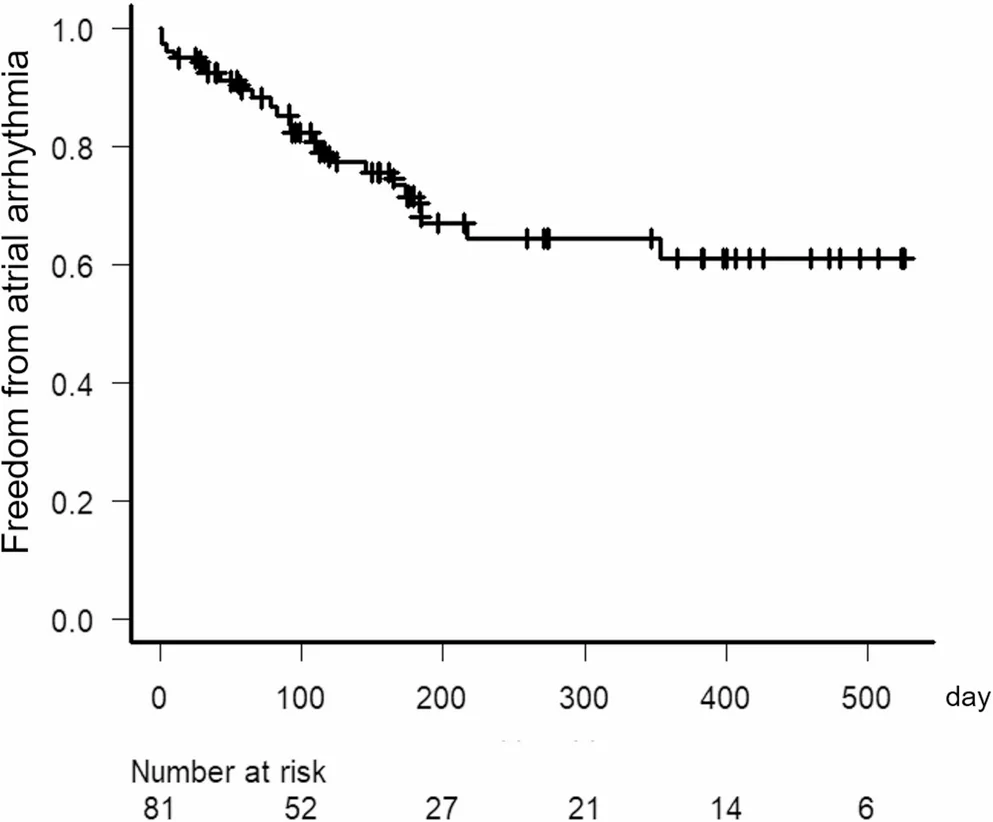
The Kaplan-Meier curve for freedom from the atrial arrhythmia recurrence after ablation without blanking period. The estimated 12-month cumulative freedom from atrial arrhythmia recurrence, including both AF or atrial tachycardia patients was 61.1% (95% confidence interval 46.1%–73.0%)

**Fig. 3:**
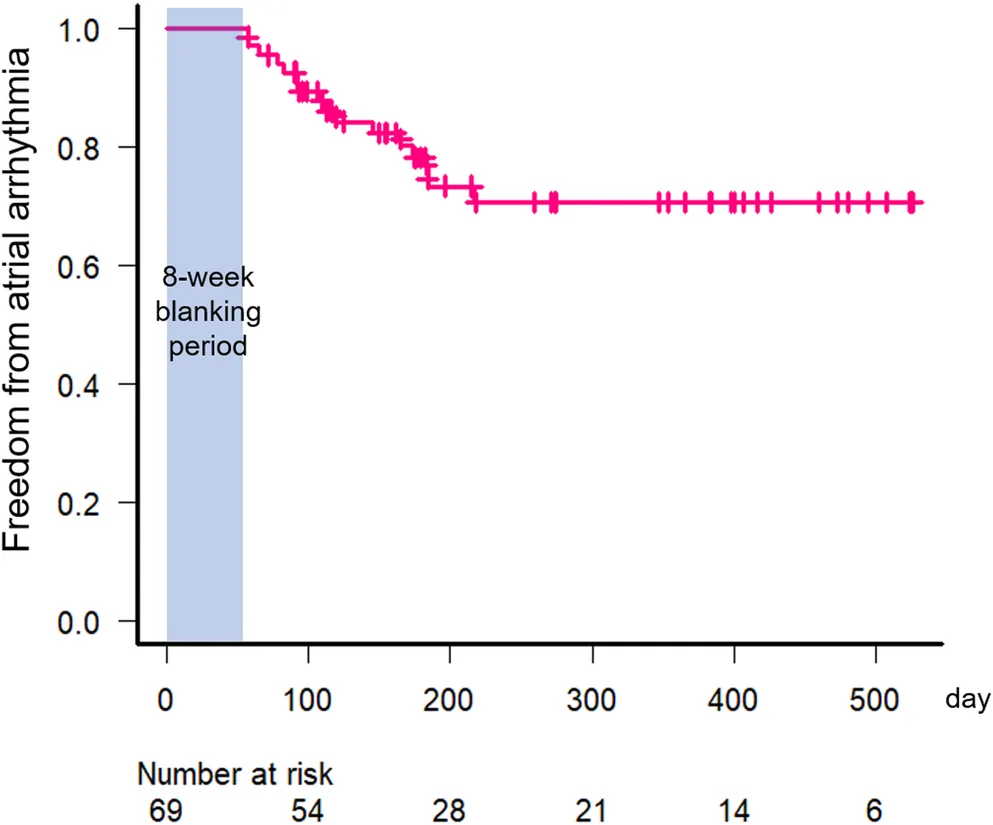
The Kaplan-Meier curve for freedom from the atrial arrhythmia recurrence after ablation after the 8-week blanking period. After the 8-week blanking period, the estimated 12-month cumulative freedom from atrial arrhythmia recurrence, including both AF or atrial tachycardia patients was 70.7% (95% confidence interval 56.0%–81.3%)

**Fig. 4:**
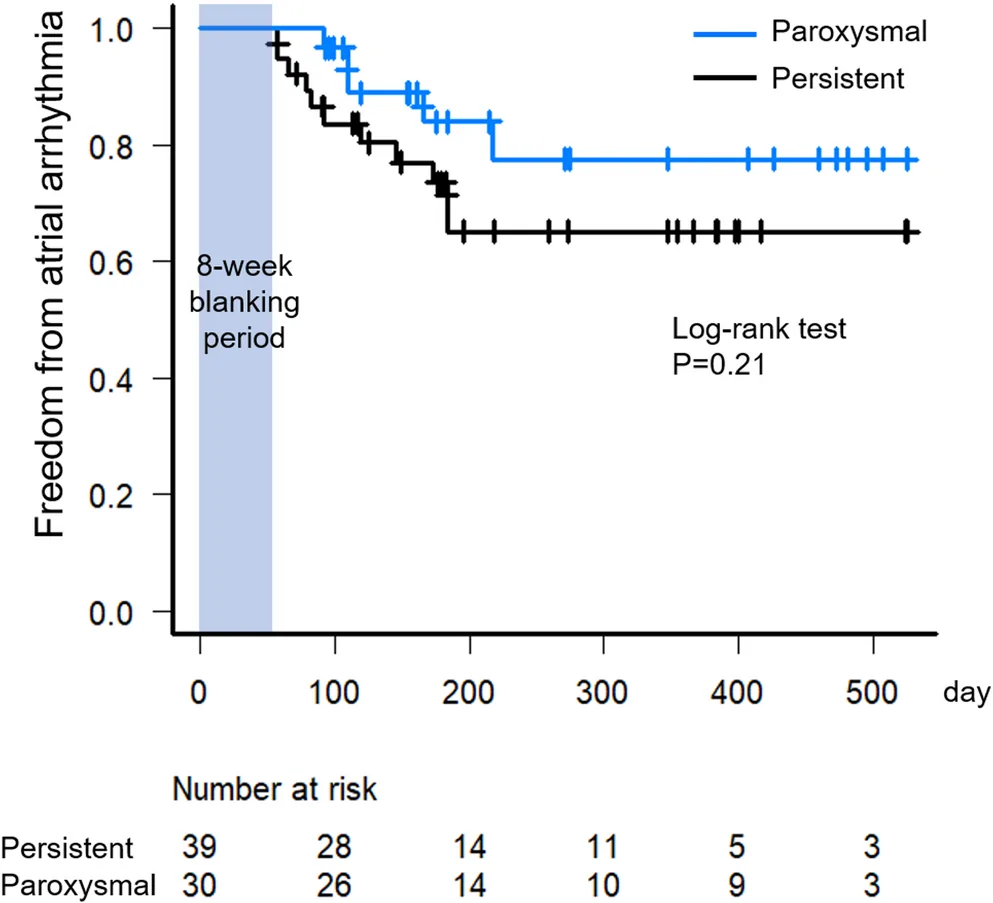
The Kaplan-Meier curve for freedom from atrial arrhythmia recurrence after ablation is shown according to paroxysmal and persistent atrial fibrillation. The recurrence rate was lower in paroxysmal atrial fibrillation (16.7%) compared to persistent atrial fibrillation (28.2%). However, the difference between the two groups was not statistically significant in the log-rank test

The mean follow-up of the overall population was 6.7 ± 5.1 months. A total of 65 patients completed 3 months, 41 completed 6 months, and 26 completed 12 months of follow-up. After this follow-up period, recurrence of any AA was observed in 23 of 81 patients (28.4%). Recurrence of AA during the blanking period was observed in five patients, of whom two experienced recurrence beyond the 8-week blanking period. Recurrence of any AA beyond the blanking period was observed in 16 of 69 patients (23.2%). The Kaplan-Meier curve for freedom from the AA recurrence after ablation is shown in Fig. 2. And, The Kaplan-Meier curve for freedom from the AA recurrence after blanking period is shown in Fig. 3. The recurrence rates for paroxysmal and persistent AF were 16.7% and 28.2%, respectively. Figure 4 shows the Kaplan-Meier curves for paroxysmal and persistent AF, with a p-value of 0.21 (Log-rank test).

### Durability of pulmonary vein isolation

Of 16 cases with AA recurrence, four patients (three atrial tachycardia, one AF) underwent a second ablation procedure. Redo procedures were performed at a median of 73 [65.3,94.8] days after the index ablation. In AT cases, PVI was intact. Among the cases of AT, one was identified as a perimitral AT, and two were related to the posterior wall. In 2 ATs related to the posterior wall, a roof line and posterior wall isolation were performed. In one patient with AF recurrence, RSPV and LSPV were reconnected and re-PVI was necessary. In redo procedures, PV reconnection was observed in 2 of 16 patients (12.5, 95%CI [1.6–38.3]).

### Procedure related complications

In our study, 1 (1.2%) patient experienced a major complication which was a stroke within 48 h post-procedure. The patient experienced sudden bilateral blurred vision with right homonymous hemianopsia, followed by transient improvement and subsequent recurrence accompanied by nausea. MRI revealed a small ischemic lesion in the border zone between the middle and posterior cerebral artery territories. The patient had no prior history of stroke and had a CHA2DS2-VA score of 1. The visual disturbance resolved promptly following admission, and no additional neurological deficits were identified. The patient was discharged without residual sequelae. The event was classified as an ischemic stroke. One patient was diagnosed with a puncture site aneurysm, which was treated conservatively and classified as a minor complication.

No patients developed electrocardiographic changes suggestive of coronary vasospasm, and no emergent coronary angiography was required. In the subset of patients who underwent pre- and post-procedural blood testing (*n* = 23), no laboratory pattern consistent with hemolysis was detected. Among biomarkers such as LDH, haptoglobin, creatinine, potassium, total and direct bilirubin, blood urea nitrogen, and reticulocyte count, only post-ablation LDH was significantly higher than the pre-ablation value (*p* < 0.01) (Supplementary Table). Additionally, no cases of postoperative phrenic nerve palsy occurred.

## Discussion

To the best of our knowledge, this is the first real world study evaluating the follow up of patients undergoing PVI with the circular multielectrode array catheter.

This study shows that:

1. Freedom from AA after PVI is 83.3% in paroxysmal and 71.8% in persistent atrial fibrillation.
2. Fourteen of sixteen pulmonary veins were still isolated.

### Efficacy of PVI using the circular multielectrode array catheter

The cumulative freedom from arrhythmia recurrence beyond the blanking period, including both AF or atrial tachycardia in the presented study was 76.8% in patients with paroxysmal or persistent AF, which is comparable to outcomes observed with radiofrequency ablation (76.9%) or cryoablation (78.6%) despite including only paroxysmal AF patients [8]. As expected, the AA recurrence rate was lower in the paroxysmal AF cohort (5 of 30 patients, 16.7%), compared to persistent AF patients (28.2%).

In the premarket study, which is so far the only study reporting outcomes using the circular multielectrode array catheter, freedom from any atrial arrhythmia recurrence after the 90-day blanking period was 69.5% and 62.3% in the paroxysmal and persistent AF cohorts, respectively [3, 6]. In other real-world reports using different pulsed field ablation catheters, the 1-year freedom from atrial arrhythmia was 74% with the Farapulse PFA ablation system (Boston Scientific, Menlo Park, CA, USA) system (80% for paroxysmal and 66% for persistent atrial fibrillation) [9]. In a separate study, the 1-year freedom from atrial arrhythmia for paroxysmal atrial fibrillation treated with the Varipulse system (Biosense Webster, Irvine, CA) was 75.4% [10]. In comparison, the outcomes of the present study demonstrated a recurrence rate comparable to these previously reported results, suggesting similar efficacy.

With respect to procedural data, our study showed a mean procedural time of 68 min, LA dwell time of 55 min, and ablation time of 29 min. Compared to the premarket clinical study (134, 65, and 58 min, respectively), all parameters were lower. However, compared to two prior real-world data studies using the circular multielectrode array catheter, our results were relatively longer (procedure time: 66.3 and 42.0 min, LA dwell time: 43.6 and 29.0 min) [11, 12], both studies did not perform 3D mapping before and after ablation in the majority of cases.

In the three observed re-do cases, 14 of 16 (87.5%) of PVs were durably isolated and three of the four patients presented with regular atrial tachycardia. Scherr et al. reported the PV reconnection rate after PFA using a pentaspline catheter, showing 28–33% for ^13^ each PV [13]. Yokoyama et al. also reported a 19.1% reconnection rate in PVI using a pentaspline PFA catheter [14]. Additionally, a meta-analysis reported a durable PVI rate per vein of 74.1% after PFA [15]. Because only four patients underwent redo ablation and these procedures were performed solely in cases with documented atrial arrhythmia recurrence, asymptomatic or unrecognized reconnection may not have been identified. Accordingly, the durability findings should be regarded as preliminary. Further studies are needed to prove if the PVI durability after ablation using the circular multielectrode array catheter will match up to these results.

### Blanking period in PFA

Recent evidence suggests a potential association between early recurrence of atrial arrhythmias (ERAA) after PFA and subsequent late recurrence (LR). Mohanty et al. reported that ERAA within the first three months following PFA occurred in 15.7% of patients and was associated with a significantly increased risk of LR [16]. Similarly, Plank et al. found that ERAA was observed in 21% of patients after PFA-PVI and served as an independent predictor of LR [17]. These findings suggest that recurrence of AA during the blanking period after PFA may be associated with subsequent recurrence, prompting ongoing discussion regarding the need to re-evaluate the duration of the blanking period in this setting. In our study, using an 8-week blanking period, early recurrence was observed in 5 patients (7.4%), of whom 2 experienced recurrence of AA beyond the blanking period.

In the SINGLE SHOT CHAMPION trial [18], recurrence rates were lower with PFA than with cryoballoon ablation; within the 3-month blanking period, atrial arrhythmia recurrence was less frequent in the PFA group, whereas thereafter, rates were similar between groups. Thus, differences in early recurrence after ablation between PFA and RF warrant careful consideration. ERAA is common after thermal ablation, often attributed to ablation-induced inflammation and autonomic imbalance, which historically justifies the use of a 90-day blanking period [19]. The substantially lower ERAA rates observed with PFA during the blanking period suggest a reduced inflammatory response, consistent with clinical observations of the low incidence of pericarditis following PFA [20]. These findings may call into question the necessity of a conventional blanking period in future PFA studies.

### Safety

PFA is believed to have the potential for lower complication rates compared to radiofrequency ablation and cryoablation. Due to the small study size of most trials investigating these new ablation tools reliable information especially on the occurrence of rare complications is not available. In our study we observed one confirmed ischemic stroke and one additional case in which a transient ischemic attack (TIA) could not be definitively diagnosed. In this stroke case, two catheter exchanges were required to perform 3D mapping, and the total left atrial dwell time reached 81 min, which is relatively prolonged despite an ablation time that remained within the standard range (34 min). ACTs during the procedure ware 305 and 276 s. Heparin was additionally administered with a target ACT of > 300 s. Pre-procedural transesophageal echocardiography performed on the same day demonstrated no evidence of left atrial thrombus. Cranial CT revealed small punctate infarcts located in the border-zone areas between the middle and posterior cerebral artery territories, without proximal arterial stenosis. This distribution is atypical for large-vessel thromboembolism or air embolism, which typically produce territorial infarctions. Instead, the pattern is more consistent with transient hypoperfusion or microembolic events. Microembolic signals detected by carotid ultrasonography and transcranial Doppler during PVI using PFA have been reported [21, 22], and PFA may be associated with silent cerebral lesions even in asymptomatic patients. Although no data indicate that PFA directly increases the incidence of clinical stroke, the presence of such silent cerebral events underscores the need for further investigation. In the latter case, transient dysarthria immediately after PVI was reported only by the patient’s husband, whereas the patient herself was unaware of any symptoms. Upon presentation, no neurological deficits were observed and cranial CT angiography revealed no acute abnormalities. Because the neurological findings were not clearly documented, a definitive diagnosis of TIA could not be established. Safety concerns regarding cerebral ischemia after PFA still cannot be excluded. Recent reports using a variable loop lasso type catheter for PFA showed an increased cerebral ischemia risk [23]. Reddy et al. reported that while asymptomatic cerebral lesions were found in 3 of 33 patients after PFA, but in none of the 37 patients after conventional thermal ablation [24]. Reichlin et al. reported a 1.0% periprocedural stroke rate with PFA [25]. In contrast, the MANIFEST-17 K registry showed a low stroke/TIA rate of 0.12% [26]. In summary the stroke risk still has to be determined for PFA PVI and there may be relevant differences between the various ablation tools.

### Application number for pulmonary vein isolation

The mean number of PFA applications per PV required for isolation was 9.8, and the LSPV required significantly more applications than the other PVs. In addition, among the non-isolated PVs that were identified, the LSPV was the most frequently observed. These findings suggest that insufficient catheter contact may occur more frequently in LSPV because of anatomical variations. Anatomical features of the LSVP—particularly the LSPV–left atrial appendage ridge—can limit catheter stability and contact when using this catheter [27]. In addition, it is possible that the design of the circular multielectrode catheter makes optimal positioning in the LSPV more difficult in some cases, which could contribute to the need for additional applications.

## Limitations

The single-center, non-randomized design and the relatively small sample size limit statistical power and generalizability. The limited number of clinical events precluded multivariable adjustment and robust subgroup analyses. Follow up is still limited in method, having been performed using holter ECG. Especially asymptomatic AF/AA episodes may have been missed and success rates therefore being overestimated. The limited intensity of rhythm monitoring may have led to an underestimation of the true recurrence rate. Rhythm follow-up relied on scheduled 24-hour Holter recordings and symptom-driven ECGs, which may underestimate the true arrhythmia recurrence rate. No control group using conventional thermal ablation technologies was included, limiting direct comparative assessment of efficacy and safety. Additionally, because of the small sample size, the statistical power of our analyses was limited, and we were unable to adjust for potential confounders. These factors restrict the strength and interpretation of the statistical findings.

## Conclusion

In this prospective real-world study, PFA using the circular multielectrode array catheter (PulseSelect Catheter) proved to be effective in both paroxysmal and persistent AF patients during long-term follow-up. Particularly in redo cases, the findings suggested a potentially favorable durability, although these observations should be interpreted as preliminary.

## Data Availability

No datasets were generated or analysed during the current study.

## Abbreviations

PFA: Pulsed field ablation
AF: Atrial fibrillation
PVI: Pulmonary vein isolation
AT: Atrial tachycardia
PV: Pulmonary vein
AA: Atrial arrhythmia
CI: Confidence interval
LA: Left atrial
ERAA: Early recurrence of atrial arrhythmias
